# Magnetic Resonance Spectroscopy Metabolites as Biomarkers of Disease Status in Pediatric Diffuse Intrinsic Pontine Gliomas (DIPG) Treated with Glioma-Associated Antigen Peptide Vaccines

**DOI:** 10.3390/cancers14235995

**Published:** 2022-12-05

**Authors:** Ashok Panigrahy, Regina I. Jakacki, Ian F. Pollack, Rafael Ceschin, Hideho Okada, Marvin D. Nelson, Gary Kohanbash, Girish Dhall, Stefan Bluml

**Affiliations:** 1 Department of Radiology, UPMC Children’s Hospital of Pittsburgh, 4401 Penn Ave Floor 2, Pittsburgh, PA 15224, USA; 2 Department of Hematology Oncology, UPMC Children’s Hospital of Pittsburgh, 4401 Penn Ave Floor 9, Pittsburgh, PA 15224, USA; 3 Department of Neurosurgery, UPMC Children’s Hospital of Pittsburgh, 4401 Penn Ave Floor 2, Pittsburgh, PA 15224, USA; 4 Department of Neurological Surgery, Box 0112 505 Parnassus Ave, University of California San Francisco, Room M779, San Francisco, CA 94143, USA; 5 Cancer Immunotherapy Program, Helen Diller Family Comprehensive Cancer Center, Box 0981 UCSF, San Francisco, CA 94143-0981, USA; 6 Department of Radiology, Children’s Hospital Los Angeles, 4650 Sunset Blvd, Los Angeles, CA 90027, USA; 7 Keck School of Medicine, University of Southern California, 1441 Eastlake Ave # 2315, Los Angeles, CA 90089, USA; 8 Department of Pediatrics, University of Alabama at Birmingham, 1600 7 th Ave S, Birmingham, AL 35233, USA

**Keywords:** brainstem glioma, MR spectroscopy, immunotherapy, pediatric brain tumor, vaccine therapy, myo-inositol, creatine, choline

## Abstract

**Simple Summary:**

Diffuse intrinsic pontine gliomas in children are rare, highly malignant infiltrating tumors in a location precluding surgical resection. The absence of non-invasive correlates of response and disease progression in these tumors invites the exploration of imaging biomarkers. Magnetic resonance spectroscopy is an advanced imaging technique for measuring cell metabolites. This study evaluated whether measurements of in vivo cell metabolites using magnetic resonance spectroscopy may serve as biomarkers of response to therapy, including progression. Single-voxel magnetic resonance spectra were serially acquired in two cohorts of patients with these tumors treated with radiation therapy with or without concurrent chemotherapy and prior to progression: 14 participants were enrolled in a clinical trial of adjuvant glioma-associated antigen peptide vaccines and 32 patients were enrolled who did not receive adjuvant vaccine therapy. In the vaccine cohort, an elevated myo-inositol/choline ratio after 2–3 doses was associated with longer survival. Scans performed up to 6 months before death showed a terminal decline in the myo-inositol/choline ratio. Higher myo-inositol/choline ratios following radiation therapy, consistent with less proliferate tumors and decreased cell turnover, were associated with longer survival, suggesting that this ratio can serve as a biomarker of prognosis following radiation therapy.

**Abstract:**

Purpose: Diffuse intrinsic pontine gliomas (DIPG) are highly aggressive tumors with no currently available curative therapy. This study evaluated whether measurements of in vivo cell metabolites using magnetic resonance spectroscopy (MRS) may serve as biomarkers of response to therapy, including progression. Methods: Single-voxel MR spectra were serially acquired in two cohorts of patients with DIPG treated with radiation therapy (RT) with or without concurrent chemotherapy and prior to progression: 14 participants were enrolled in a clinical trial of adjuvant glioma-associated antigen peptide vaccines and 32 patients were enrolled who did not receive adjuvant vaccine therapy. Spearman correlations measured overall survival associations with absolute metabolite concentrations of myo-inositol (mI), creatine (Cr), and *n*-acetyl-aspartate (NAA) and their ratios relative to choline (Cho) during three specified time periods following completion of RT. Linear mixed-effects regression models evaluated the longitudinal associations between metabolite ratios and time from death (terminal decline). Results: Overall survival was not associated with metabolite ratios obtained shortly after RT (1.9–3.8 months post-diagnosis) in either cohort. In the vaccine cohort, an elevated mI/Cho ratio after 2–3 doses (3.9–5.2 months post-diagnosis) was associated with longer survival (rho = 0.92, 95% CI 0.67–0.98). Scans performed up to 6 months before death showed a terminal decline in the mI/Cho ratio, with an average of 0.37 ratio/month in vaccine patients (95% CI 0.11–0.63) and 0.26 (0.04–0.48) in the non-vaccine cohort. Conclusion: Higher mI/Cho ratios following RT, consistent with less proliferate tumors and decreased cell turnover, were associated with longer survival, suggesting that this ratio can serve as a biomarker of prognosis following RT. This finding was seen in both cohorts, although the association with OS was detected earlier in the vaccine cohort. Increased mI/Cho (possibly reflecting immune-effector cell influx into the tumor as a mechanism of tumor response) requires further study.

## 1. Introduction

Diffuse intrinsic pontine gliomas (DIPG) in children are rare, highly malignant infiltrating tumors in a location precluding surgical resection. The median overall survival (OS) is less than one year, and no significant improvement in outcome has been achieved for decades [1]. Radiation therapy remains the only therapy with demonstrated short-term clinical benefit [2]. Although the overall prognosis is extremely poor, some patients have a longer reprieve after completing radiation therapy before the almost inevitable recurrence. Molecular biology studies have shown that certain patterns of gene mutations, most commonly involving histone H3 genes and other loci, may in part account for this variability [3,4], but this remains controversial.

The absence of non-invasive correlates of response and disease progression in DIPG invites the exploration of imaging biomarkers. Magnetic resonance spectroscopy (MRS) is an advanced imaging technique for measuring cell metabolites and can be readily integrated into standard clinical magnetic resonance (MR) imaging protocols. Cell metabolite profiles in newly diagnosed patients are often more characteristic of low-grade tumors, which may reflect that infiltrating tumor cells are interspersed with normal structures; as DIPG progresses after radiation therapy, metabolic profiles become increasingly consistent with higher-grade lesions, potentially reflecting the predominance of malignant cells in the tumor [5,6]. For example, increasing choline (Cho) typically indicates increasing membrane turnover and cell proliferation, which can be seen in fast-growing tumors as well as in inflammatory processes. Absolute or relative levels of other metabolites reflect the tumor physiology seen during malignant progression, e.g., decreased energy stores reflected by creatine (Cr), decreased astrocytic cell markers reflected by myo-inositol (mI), and decreased markers of healthy axons and neurons (*n*-acetyl aspartate, NAA) [7,8,9,10,11,12]. 

Our recently reported peptide vaccine study [13] targeted multiple glioma-associated antigen (GAA) epitopes as adjuvant therapy for children with malignant brain tumors, including a cohort of children with DIPG. Interferon-γ enzyme-linked immunosorbent spot (ELISPOT) assays identified immune responses to GAA epitopes with considerable between-patient variation in epitope response magnitude and duration. The therapy was generally well-tolerated, and the median OS (nominally superior to historical controls) in this highly selected cohort was encouraging. We performed MRS on a subset of patients with DIPG prior to and following the start of vaccine therapy. In a cohort of patients with DIPG from a separate institution who did not receive immunotherapy, MRS was performed prior to and following RT. Our hypothesis was that MRS indicators of progression would be slower or delayed in patients with longer overall survival, supporting further development of MRS as a non-invasive biomarker for DIPG treatment response and progression. 

## 2. Materials and Methods

### 2.1. Patients and MR Spectroscopy 

Informed consent was obtained for all patients enrolled in the peptide vaccine therapy clinical trial (ClinicalTrials.gov #NCT01130077), in accordance with institutional review board (IRB) policies. The MR spectroscopy was performed as part of the clinical MRI protocol. All patients completed irradiation (RT) with or without concurrent chemotherapy. Due to the detrimental impact of steroids on the effectiveness of immunotherapy, patients had to be off or on a very low dose of dexamethasone prior to trial enrollment. Additional details about trial inclusion and treatment may be found in the primary publication [13]. Parental consent was obtained for patients in the non-vaccine cohort who were enrolled in a number of prospective clinical trials, most of which involved irradiation with concurrent chemotherapy and adjuvant non-immunotherapy. An IRB waiver of consent was obtained to examine data acquired as part of the clinical care for patients who were not enrolled in a clinical trial. Thirteen of the thirty-two patients in the non-vaccine cohort were also included in a prior report [6]. 

For participants in the vaccine trial, vaccines were administered every 3 weeks x 8, and MRI scans were obtained at the baseline, weeks 6, 15, and 24, and then every 12 weeks or as clinically indicated. MRS studies were obtained between the pre- and post-contrast MR imaging. However, MRS studies were not obtained on every patient or at the time of each MRI. All studies were conducted on a clinical 1.5 T MR system (Signa LX, GE Healthcare, Milwaukee, WI, USA). Single-voxel point-resolved spectroscopy (PRESS) with a short echo time (TE) of 35 ms, a repetition time (TR) of 1.5 s, and 128 signal averages was used for all acquisitions. Sizes and shapes of the ROIs were adjusted to lesion size and typically varied between 5 and 10 cm^3^. The total acquisition time, including scanner adjustments, was less than five minutes per spectrum. Spectra were centrally processed with fully automated LCModel (Stephen Provencher Inc., Oakville, Ontario, Canada, LCModel Version 6.3–1 L) software [14]. T2-weighted fast spin-echo, FLAIR, and T1-weighted FLAIR images were acquired in all cases, and the position of the region of interest (ROI) was documented on at least three MR images. All ROIs were a priori and independently reviewed to ensure that only spectra that were consistently positioned and representative of the tumor tissue were retained for subsequent processing and analysis.

Evaluation of MRS data was limited to metabolite concentrations and metabolite ratios deemed reliable as per previously published studies [5,6,9,10,11,12] (i.e., choline (Cho), creatine (Cr), myo-inositol (mI), *n*-acetyl-aspartate (NAA), and lactate (Lac)). Absolute metabolite levels were determined by using the unsuppressed water signal as the reference signal and the default water content set by the LCModel software (65% ≈ 36.1 mol/kg). The relative concentrations of mI/Cho, Cr/Cho, and NAA/Cho were the primary endpoints rather than absolute concentrations to reflect underlying processes involving multiple metabolites and to control for possible scan-level factors. Lipid intensities (with possible contributions from underlying macromolecules) at 1.3 ppm (from -CH_2_ groups of lipid molecules, LipMM13) and at 0.9 ppm (terminal -CH_3_, LipMM09) were analyzed as indicators of progressive disease [15]. 

### 2.2. Statistical Analysis

To identify MRS biomarkers associated with overall survival at predefined timepoints, scans for both cohorts (vaccine and non-vaccine) were binned into comparable timepoints defined by the vaccine therapy schedule (vaccine cohort). Timepoint 0 (time of diagnosis, pre-RT) scans were obtained only in the non-vaccine cohort. Timepoint 1 was post-RT and prior to vaccine therapy (1.9–3.8 months post-diagnosis) (vaccine cohort and non-vaccine cohort). Timepoint 2 included scans obtained after 2–3 vaccine doses (3.9–5.2 months post-diagnosis) (vaccine cohort and non-vaccine cohort) and timepoint 3 scans were performed after 4–6 vaccine doses (5.3–7.6 months post-diagnosis) (vaccine cohort and non-vaccine cohort). Spearman rank-order correlations were used to measure associations between timepoint-specific metabolite ratios and overall survival, with confidence intervals estimated using Fisher’s r-to-z transformation. A single generalized estimating equations model for each ratio was considered instead of Spearman correlations, to adjust for multiple records from a single subject in the same timepoint and across time. The two cohorts were analyzed separately without direct comparison of associations between metabolites and survival due to limitations with the sample size and missing data points. The false discovery rate for groups of correlations was controlled at 0.05 through the method of Benjamini and Hochberg [16]. For a separate analysis summarizing longitudinal trends based on the time from death rather than the time from diagnosis, linear mixed-effects (random intercept) regression was used to evaluate the terminal decline in metabolite values and ratios. Statistical analyses were performed using SAS/STAT statistical software version 9.4 (SAS Institute, Cary, NC, USA) and R version 4.0.2 (R Foundation for Statistical Computing, Vienna, Austria).

## 3. Results

**Figure 1:**
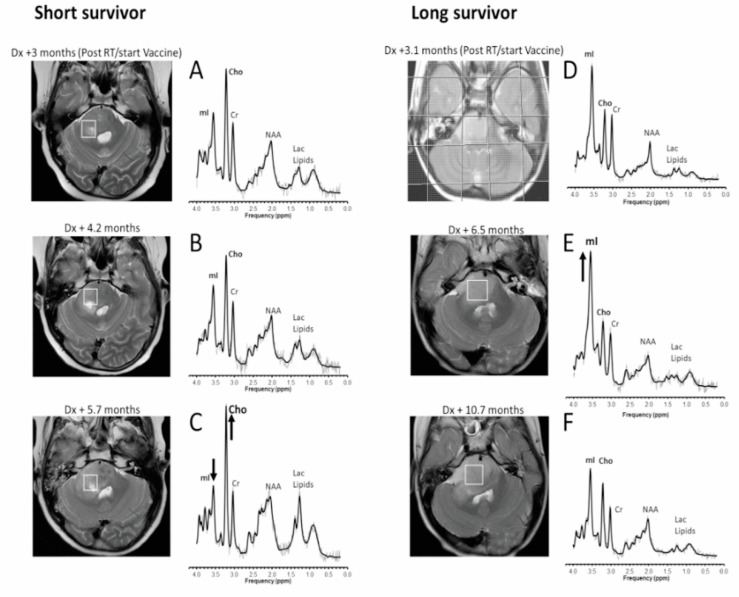
Transverse T2-weighted MRI and corresponding MR spectra. For a patient who survived 8.5 months after diagnosis (left column), metabolite levels at the start (**A**) and 1.5 months into vaccine therapy (**B**) are comparable. Soon before clinical and radiological deterioration (**C**), myo-inositol (mI) declined, and choline (Cho), lactate (Lac), and lipids all increased. For a patient who survived 23.6 months after diagnosis (**right column**), mI was prominent after completion of radiotherapy (**D**), and a further increase relative to Cho was noticeable 3 months after start of vaccine therapy (**E**). After another 4.2 months (and over a year before death) (**F**), a gradual decline of mI and a gradual increase in Cho were observed. Dx = diagnosis, RT = irradiation with or without concurrent chemotherapy, mI = myo-inositol, Cho = choline, Cr = creatine, Lac = lactate. Shown are the unfiltered raw data (thin line) and the fit to the data used for quantitation (thick line).

Fifty-eight MRS studies (1–8 scans per patient, median 4) were performed in 14 patients with DIPG enrolled in the peptide vaccine study. Eighty-two MRS studies (1–7 scans per patient, median 2.5) were performed on 32 patients in the non-vaccine cohort. Table 1 shows patient characteristics for the vaccine cohort (diagnosed between 2010 and 2012) and the non-vaccine cohort (diagnosed between 2001 and 2014). Patients were aged 2.2 to 17.9 years at diagnosis, with a median overall survival from diagnosis of 13.5 months (range 6.3–23.6 months) for the vaccine cohort and 11.2 months (range 3.7–37.6 months) for the non-vaccine cohort. The example spectra for patients with short survival and long survival are shown in Figure 1.

**Figure 2:**
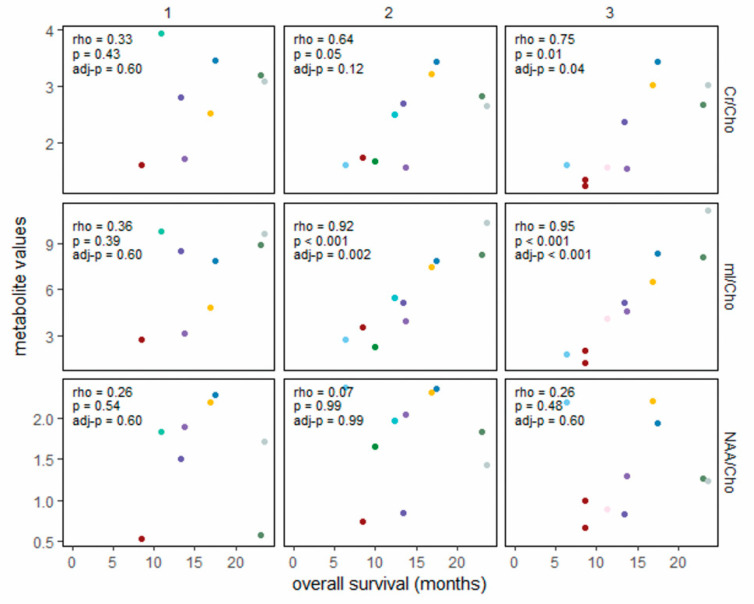
Association between metabolite ratios and overall survival in the vaccine cohort, grouped by timepoints relative to completion of RT (columns) (28 scans within 7.6 months of diagnosis in 12 patients). RT = irradiation with or without concurrent chemotherapy. Spearman rank-order correlations are shown for each panel, with Benjamini–Hochberg control of *p*-values to a false positive rate of 0.05 for all panels. Each color represents an individual patient. Timepoint 1 = post-RT and before vaccine therapy (1.9–3.8 months post-diagnosis); timepoint 2 = after 2–3 vaccine doses (3.9–5.2 months post-diagnosis); timepoint 3 = after 4–6 vaccine doses (5.3–7.6 months post-diagnosis).

**Figure 3:**
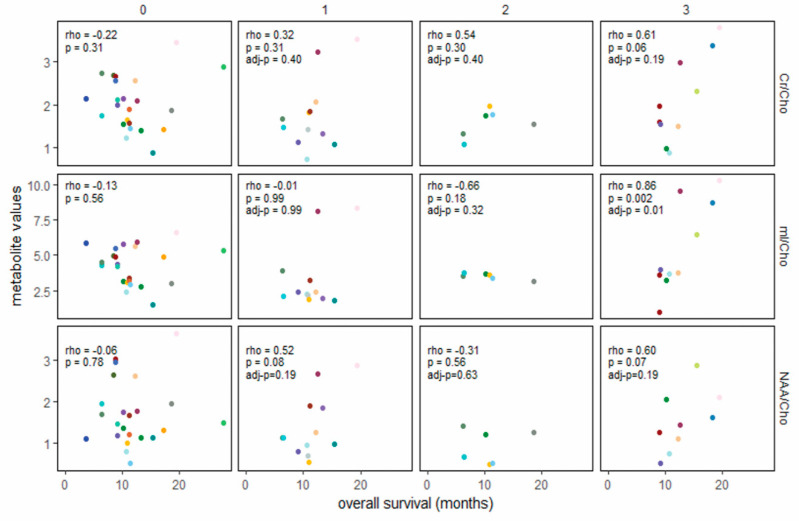
Association between metabolite ratios and overall survival in the non-vaccine cohort, at diagnosis (*n* = 23, left column), and at 3 timepoints defined relative to date of diagnosis to align with dosing categories for the vaccine cohort (28 scans within 7.6 months of diagnosis in 19 patients). RT = irradiation with or without concurrent chemotherapy. Spearman rank-order correlations are shown for each panel, with Benjamini–Hochberg control of *p*-values to a false positive rate of 0.05 for timepoints 1–3. Each color represents an individual patient. 0 = diagnosis, before RT; 1 = 1.9–3.8 months post-diagnosis (post-RT for most); 2 = 4.0–5.2 months post-diagnosis; 3 = 5.3–7.6 months post-diagnosis.

Figure 2 shows associations in the vaccine cohort between metabolite ratio values within predefined time periods (defined above, timepoints 1–3) and survival duration. Figure 3 shows the same associations for the non-vaccine cohort, with an additional column for measurements obtained at the time of diagnosis (timepoint 0, pre-RT). At diagnosis, the ratios of interest (mI/Cho, Cr/Cho, and NAA/Cho) did not correlate with overall survival (first column of Figure 3). The ratios following RT (1.9–3.8 months post-diagnosis) also did not appear to be associated with overall survival in either cohort. By 3.9–5.2 months post-diagnosis (after 2–3 vaccine doses in the vaccine cohort), mI/Cho in the vaccine cohort was strongly associated with overall survival (rho = 0.92, 95% CI 0.67–0.98), even though the range of mI/Cho values was similar to the range at timepoint 1. The six patients scanned within the same timeframe in the non-vaccine cohort showed very little variation in mI/Cho values, precluding an association with overall survival. At 5.3–7.6 months post-diagnosis, the mI/Cho values in the vaccine cohort still showed a strong rank-order correlation with survival (rho = 0.95, 95% CI 0.81–0.99), a positive association that was also seen in the non-vaccine cohort (rho = 0.86, 95% CI 0.49–0.97). Descriptive displays of each patient’s series of metabolite ratios and values relative to the time of diagnosis are shown in Figures S1–S4 to support the analyses presented above. 

These associations of mI/Cho with overall survival at timepoints 2 and 3 (3.9–7.6 months post-diagnosis) demonstrate promise for mI/Cho as a non-invasive biomarker for the prognosis at those timepoints. However, co-variation in metabolite values and survival at these timepoints may reflect the influence of treatment response or terminal decline, or potentially a combination of both. As an exploratory analysis, to isolate the effects of terminal change, we present a separate analysis counting time backward from death instead of forward from diagnosis. The MR spectra of vaccine cohort patients demonstrated a terminal decline of the three metabolite ratios, most clearly observed for mI/Cho (Figure S5). The rate of decline was estimated using random intercept linear mixed models of scans obtained within 6 months of death (Table 2). In the vaccine cohort, the mI/Cho ratio decreased an average of 0.37 units (95% CI 0.11–0.63)/month before death (*p* = 0.01). The average decrease in Cr/Cho was 0.12 units/month (95% CI 0.05–0.19, *p* = 0.003), and the average decrease in NAA/Cho was 0.06 units/month (95% CI −0.06–0.18, *p* = 0.27). No individual patient’s data were unduly influential regarding the slope estimates; comparable slopes were obtained for models with both random slope and intercept and when analyzing scans within 12 months rather than within 6 months of death (Table 2). All three metabolite ratios also showed a pattern of terminal decline in the non-vaccine cohort, with attenuated slopes for mI/Cho and Cr/Cho compared to the vaccine cohort (Table 2). Terminal decline trajectories for individual metabolites are shown in Figure S6 (vaccine cohort) and Figure S7 (non-vaccine cohort). Myo-inositol showed a terminal decline for most patients in the vaccine cohort, with an average decrease of 0.92 i.u./month (95% CI 0.28–1.56, *p* = 0.009) in the last 6 months before death. High lactate and lipid levels were observed in some patients within months of death. 

## 4. Discussion

Diffuse intrinsic pontine gliomas are incurable pediatric brain tumors, with most patients succumbing to their disease within a year of diagnosis. Recently, peptide vaccines and other immunotherapies have been developed as experimental treatments for these tumors [13,17]. Tumors in the pons cannot feasibly undergo serial biopsies to monitor their status; non-invasive means of monitoring response to treatment are needed. We reviewed the in vivo metabolic profiles of two cohorts of DIPG patients: participants in a clinical trial of peptide vaccine therapy (ClinicalTrials.gov #NCT01130077) and patients from a different institution who did not receive immunotherapy, to evaluate possible biomarkers of disease status. High mI/Cho ratios at 3.9–5.2 months following diagnosis, corresponding to the time frame when patients on the vaccine trial had received 2–3 vaccine doses, were associated with longer survival (Figure 2 and Figure S1). This pattern was also seen in the non-vaccine cohort, although the association between higher mI/Cho ratios and longer overall survival was not observed until later (5.3–7.6 months post-diagnosis) (Figure 3 and Figure S2).

Declines in mI/Cho, NAA/Cho, and Cr/Cho over time in both the vaccine cohort and non-vaccine cohort were observed (Table 2). First, we define benchmark timepoints for assessing MRS biomarkers to support their development for clinical use as a non-invasive prospective biomarker for disease status. Additionally, this study provides data to enhance the understanding of potential mechanisms of therapy response and disease progression by (a) presenting data from two separate cohorts: a clinical trial evaluating adjuvant immunotherapy and an independent cohort of patients treated with (chemo)radiation but not immunotherapy; and (b) examining MRS patterns forward from the time of diagnosis and backward from the time of death. Prior studies emphasized high or increasing Cho, low or decreasing NAA/Cho or Cr/Cho, and/or high lactate and lipids as indicators of poor prognosis [5,6,18,19,20,21]. While we found support for these findings in selected cohorts (Table 2) or patient trajectories (Figures S3 and S4), the pattern displayed most consistently across both cohorts was a terminal change in mI/Cho (Table 2, Figure S5). 

Our study is one of the first to show a potentially useful non-invasive biomarker that can be evaluated after the post-RT MRI scan in patients with DIPG. Most DIPG neuroimaging biomarker studies have focused on the relationship between tumor volume and outcomes before radiation therapy [12,22]. These studies have demonstrated that the MRI response after completion of RT in comparison to the size pre-RT can be prognostic. For example, in patients receiving RT for DIPG, the largest decrease in tumor size was found at 2 weeks following completion of RT, with minimal subsequent changes observed in imaging at 6 to 8 weeks post-RT [23]. Other studies have shown that a >25% decrease in tumor volume following RT is significantly associated with better overall survival [24]. Further studies are needed to determine if there is an association between the mI/Cho ratio at this such timepoint and the change in the size of the tumor. Increased relative or absolute choline concentrations are believed to indicate increased cell membrane metabolism, and decreasing NAA is believed to reflect the replacement of residual axons/neurons by infiltrative tumor cells [10]. Lipids and lactate levels have been shown to increase as degenerating tumors outgrow their perfusion support and develop areas of necrosis [25].

Increasing mI in brain tumors is considered to reflect an increase in reactive astrocytes, microglia, and/or gliosis, either as an effect of radiation therapy or chronic inflammation. Similarly, low levels of choline can also reflect inactive glial cells. Other studies have suggested the converse, that decreasing mI and/or increasing choline in DIPG were consistent with progressive disease/reactivation of tumors [5,6,11], with the decrease in mI potentially a result of increasing tumoral edema and the increased choline reflective of increased cell turnover. The strong association of high mI/Cho ratios seen after 2–3 vaccines and sustained over a few months in patients with longer-term survival in vaccine therapy could be due to infiltration by vaccine-responsive immune cells with resultant inflammation and proliferation of microglia. In the non-vaccine cohort, the increase in mI/Cho occurred slightly later after the completion of RT, potentially reflecting a later onset of gliosis. This hypothesis is supported by consistently high mI in the enhancing brain lesions seen in multiple sclerosis [26,27,28] and the surrounding white matter [29,30], a disease primarily caused by T-lymphocyte infiltration with resultant activation and proliferation of microglia. 

DIPG are relatively rare tumors, and the number of patients included in the early phase vaccine study is small, resulting in several study limitations. Because patients could complete initial RT before traveling to the vaccine trial study site, MRS data were not acquired at diagnosis or during RT for that cohort, so we were not able to evaluate previously reported prognostic factors at this timepoint [21]. While MRS measures describe a relevant range of tumor characteristics, targeted immune-imaging tracers [31,32] may also play a role in the development and selection of DIPG patients for immunotherapy trials. The small sample size also precluded the examination of MRS measures in patients with pseudoprogression (transient increased edema and/or contrast enhancement of the tumor, followed by stabilization/regression and symptomatic improvement), which was postulated to be an indicator of the vaccine treatment efficacy [13,33]. Finally, while steroids are relatively contra-indicated during vaccine therapy due to the suppression of an immune response, steroids are generally part of the initial management of symptoms as well as at the time of any neurologic worsening. It is unclear whether and to what extent steroids have an impact on metabolite concentrations and their measurement via MR spectroscopy. Although the imaging protocol was the same for both cohorts, differences in referral patterns to the sites, treatment regimens, small numbers of patients, and differences in scan timing introduce unmeasured confounders into treatment cohort comparisons, limiting direct comparisons. 

## 5. Conclusions

In summary, long-term survivors were characterized by high mI/Cho ratios that developed following initial RT, suggesting that mI may constitute an in vivo, real-time surrogate indicator for the tumor microenvironment response to RT. The number of patients with serial MRS evaluations on the vaccine trial was too small to draw strong conclusions about the role of the vaccine in the observation of an increased mI/Cho ratio slightly earlier than in the non-immunotherapy cohort (Figure 2 and Figure 3). The six-month terminal decline observed in 10 patients receiving peptide vaccine therapy and 18 patients in a non-vaccine cohort supports the hypothesis that progressive DIPG is characterized by increasing tumor membrane turnover (choline) accompanied by decreasing mI and creatine. Although it is possible that the more distinctive terminal decline slopes in the vaccine study (Table 2, Figure S5) are related to mechanisms of vaccine therapy resistance, the more likely explanation (beyond chance variation) is the uniformity of the vaccine therapy regimen compared to the non-vaccine cohort. These remain to be validated in other cohorts receiving RT with and without immunotherapy. Increased mI/Cho (possibly reflecting immune-effector cell influx into the tumor as a mechanism of tumor response) requires further study.

## Figures and Tables

**Table 1:** Patient characteristics.

	Vaccine Cohort (*n* = 14)	Non-Vaccine Cohort (*n* = 32)
	Median (range)	Median (range)
Age at diagnosis, years	8.5 (2.2–17.9)	7 (3–15)
Number of MRS per patient	4 (1–8)	2.5 (1–7)
Survival from diagnosis, months	13.5 (6.3–23.6)	11.2 (3.7–37.6)
Sex		
Female	6 (43)	19 (59)
Male	8 (57)	13 (41)
Pre-vaccine therapy		
Radiotherapy only	10 (72)	6 (19)
Radiotherapy + bevacizumab	2 (14)	1 (3)
Radiotherapy + temozolomide	2(14)	5 (16)
Radiotherapy + unknown/other *		20 (62)

MRS = magnetic resonance spectroscopy; * includes carboplatin + etoposide, gadolinium-texaphyrin, temozolomide + irinotecan.

**Table 2:** Monthly rate of terminal decline for key metabolite ratios, excluding scans at diagnosis. Linear mixed-effects (random intercept) models for each metabolite, fit separately for each study cohort.

	Within 6 Months of Death		Within 12 Months of Death	
	Vaccine Cohort (*n* = 22 Scans in 10 Patients)	Non-Vaccine Cohort (*n* = 37 Scans in 18 Patients)	Vaccine Cohort (*n* = 44 Scans in 13 Patients)	Non-Vaccine Cohort (*n* = 59 Scans in 23 Patients)
**Metabolite ratio**	Slope (95% CI)	Slope (95% CI)	Slope (95% CI)	Slope (95% CI)
**mI/Cho**	0.37 (0.11–0.63)	0.26 (0.04–0.48)	0.37 (0.21–0.54)	0.19 (0.05–0.34)
**Cr/Cho**	0.12 (0.05–0.19)	0.06 (-0.02–0.13)	0.11 (0.06–0.17)	0.06 (0.02–0.10)
**NAA/Cho**	0.06 (−0.06–0.18)	0.02 (−0.06–0.11)	0.07 (0.02–0.11)	0.08 (0.04–0.12)

mI = myo-inositol, Cho = choline, Cr = creatine.

## Data Availability

Preliminary results have been presented at the 2015 Clinical Translation Pediatric Neurooncology symposium, in a 2017 publication describing advanced MR imaging in the peptide vaccine study [34], and at the 2019 International Society for Magnetic Resonance in Medicine annual meeting. The data presented in this study are available on request from the corresponding author. The data are not publicly available due to patient privacy.

## Supplementary Materials

The following supporting information can be downloaded at: https://www.mdpi.com/article/10.3390/cancers14235995/s1, Figure S1: Longitudinal trajectories (relative to date of diagnosis) for metabolite ratios of interest, Figure S2: Longitudinal trajectories (relative to date of diagnosis) for metabolite ratios of interest, Figure S3: Longitudinal trajectories (relative to date of diagnosis) for individual metabolites, Figure S4: Prospective change (relative to date of diagnosis) for individual metabolites, Figure S5: Terminal decline (relative to date of death, X = 0) for metabolite ratios of interest measured within 12 months of death, excluding scans at diagnosis: myo-Inositol/choline (left column), creatine/choline (middle), NAA/choline (right column). Figure S6: Terminal change (relative to date of death) for individual metabolites (mmol/kg), Figure S7: Terminal change (relative to date of death) for individual metabolites (mmol/kg).
