# Supplementary material for: Magnetic Resonance Spectroscopy Metabolites as Biomarkers of Disease Status in Pediatric Diffuse Intrinsic Pontine Gliomas (DIPG) Treated with Glioma-Associated Antigen Peptide Vaccines
Source: Cancers (Basel). 2022 Dec 5;14(23):5995. doi: 10.3390/cancers14235995 (PMC9739009; doi:10.3390/cancers14235995)
Supplement: Supplementary file 1 [file cancers-14-05995-s001.zip › cancers-1928640-supplementary.pdf]

Magnetic Resonance Spectroscopy Metabolites as Biomarkers of Disease Status in Pediatric Diffuse Intrinsic Pontine Gliomas (DIPG) treated with Glioma-Associated Antigen Peptide Vaccines

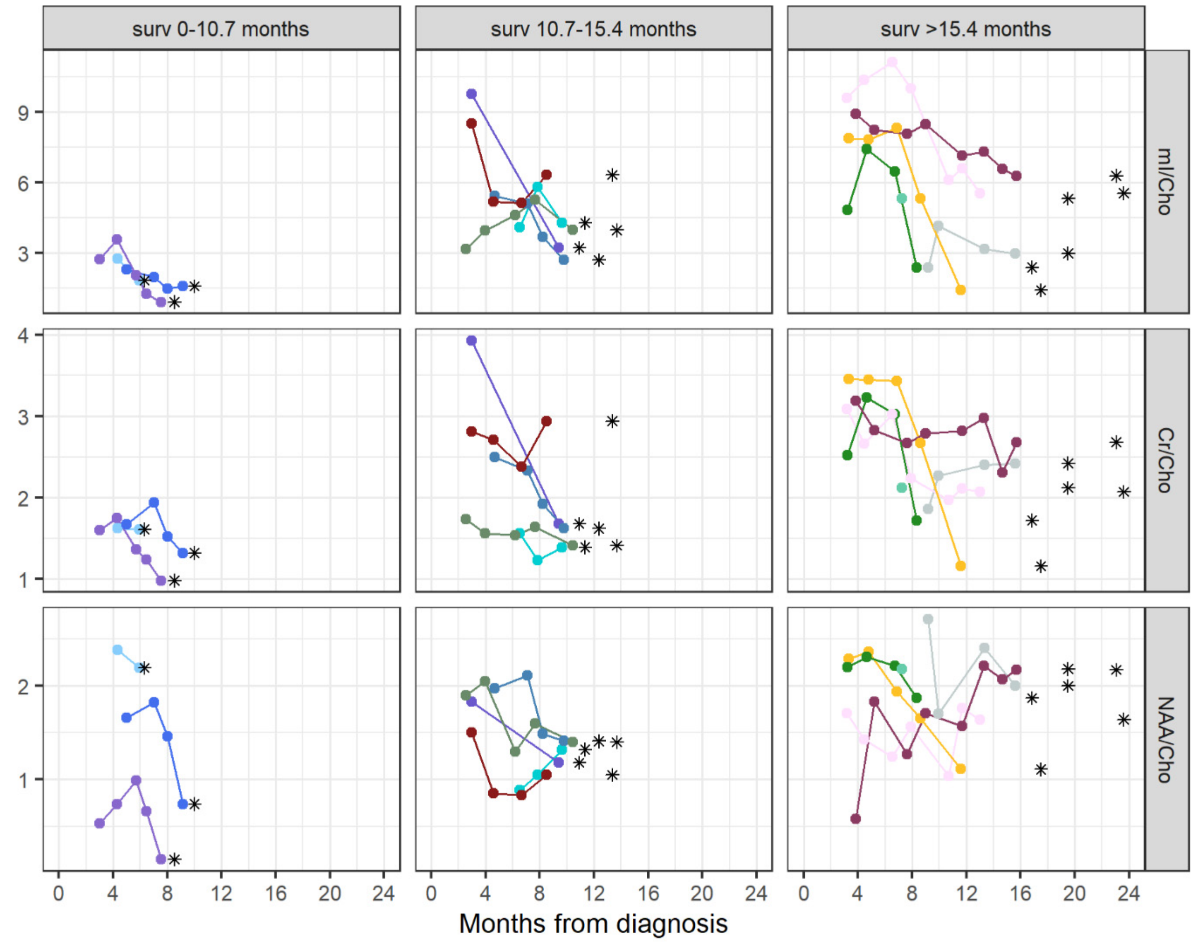

Figure S1. Longitudinal trajectories (relative to date of diagnosis) for metabolite ratios of interest, **Vaccine Cohort** (58 scans in 14 patients). Each patient appears in one of three columns, defined as tertiles of pooled overall survival from the vaccine study and non-vaccine cohort, with 10.7 months coinciding approximately with the median survival for patients treated with irradiation with or without concurrent chemotherapy.{Clymer, 2018 #30;Renfro, 2019 #56}. An immediate terminal decline of mI/Cho is observed in shorter surviving patients. x denotes the time of death, placed for each patient at Y-value of final observed metabolite ratio. *Absolute metabolite levels in above supplemental figure and all following figures are in institutional units (u.i.).*

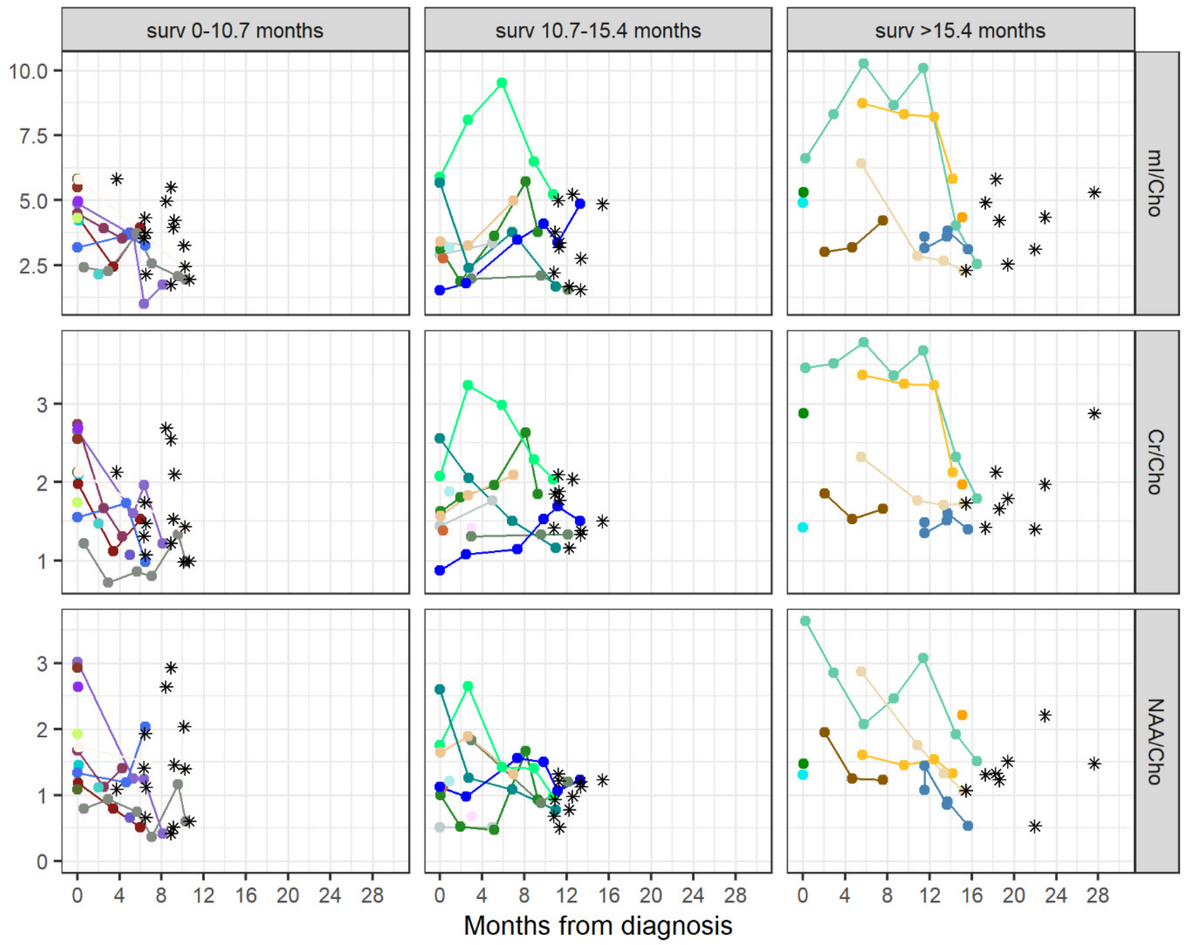

Figure S2. Longitudinal trajectories (relative to date of diagnosis) for metabolite ratios of interest, **Non-vaccine Cohort** (87 scans in 32 patients). Each patient appears in one of three columns, defined by survival (surv) duration. An immediate terminal decline of mI/Cho is observed in shorter surviving patients. x denotes the time of death, placed for each patient at the Y-value of the final observed metabolite ratio. .

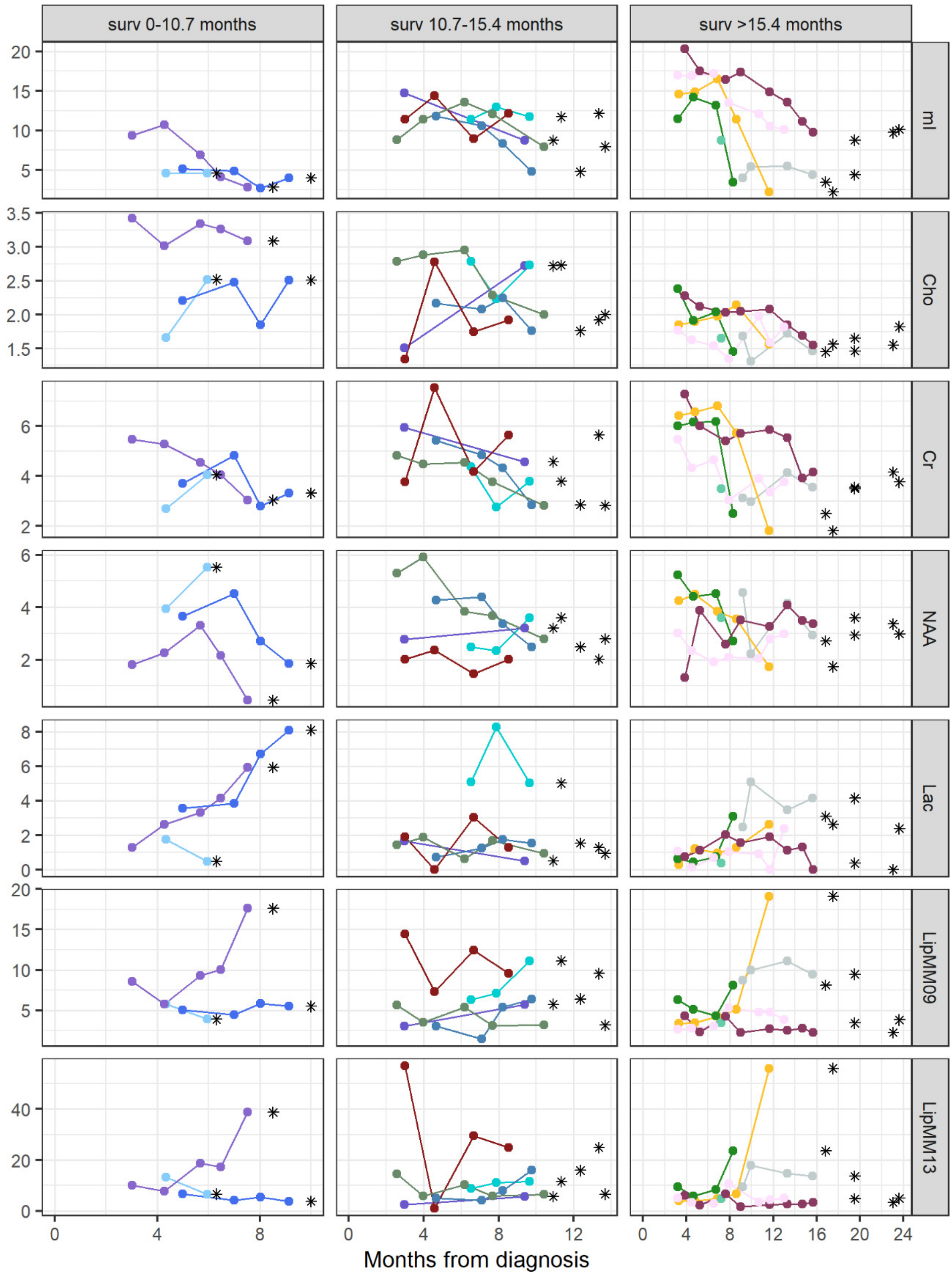

Figure S3. Longitudinal trajectories (relative to date of diagnosis) for individual metabolites, **Vaccine Cohort** (58 scans in 14 patients, see Figure S1 for key). Each row contains values for a metabolite. The three columns sort patients by post-diagnosis survival. **x** = time of death on X axis, with Y axis value the last measured metabolite value.

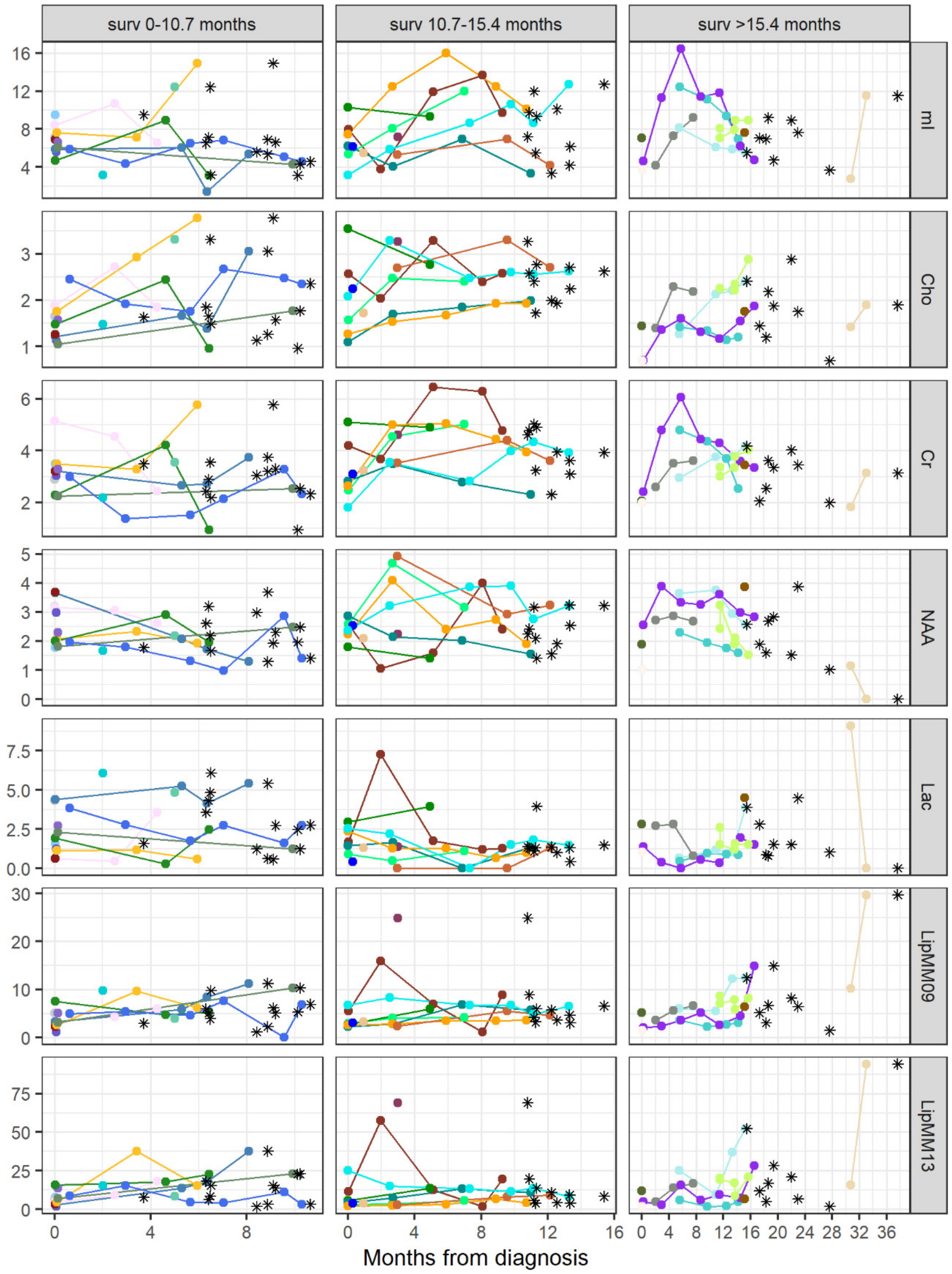

Figure S4. Prospective change (relative to date of diagnosis) for individual metabolites, **Non-vaccine Cohort** (87 scans in 32 patients, see Figure S5 for key). Each row contains values for a metabolite. The three columns sort patients by post-diagnosis survival. **x** = time of death on X axis, with Y axis value the last measured metabolite value.

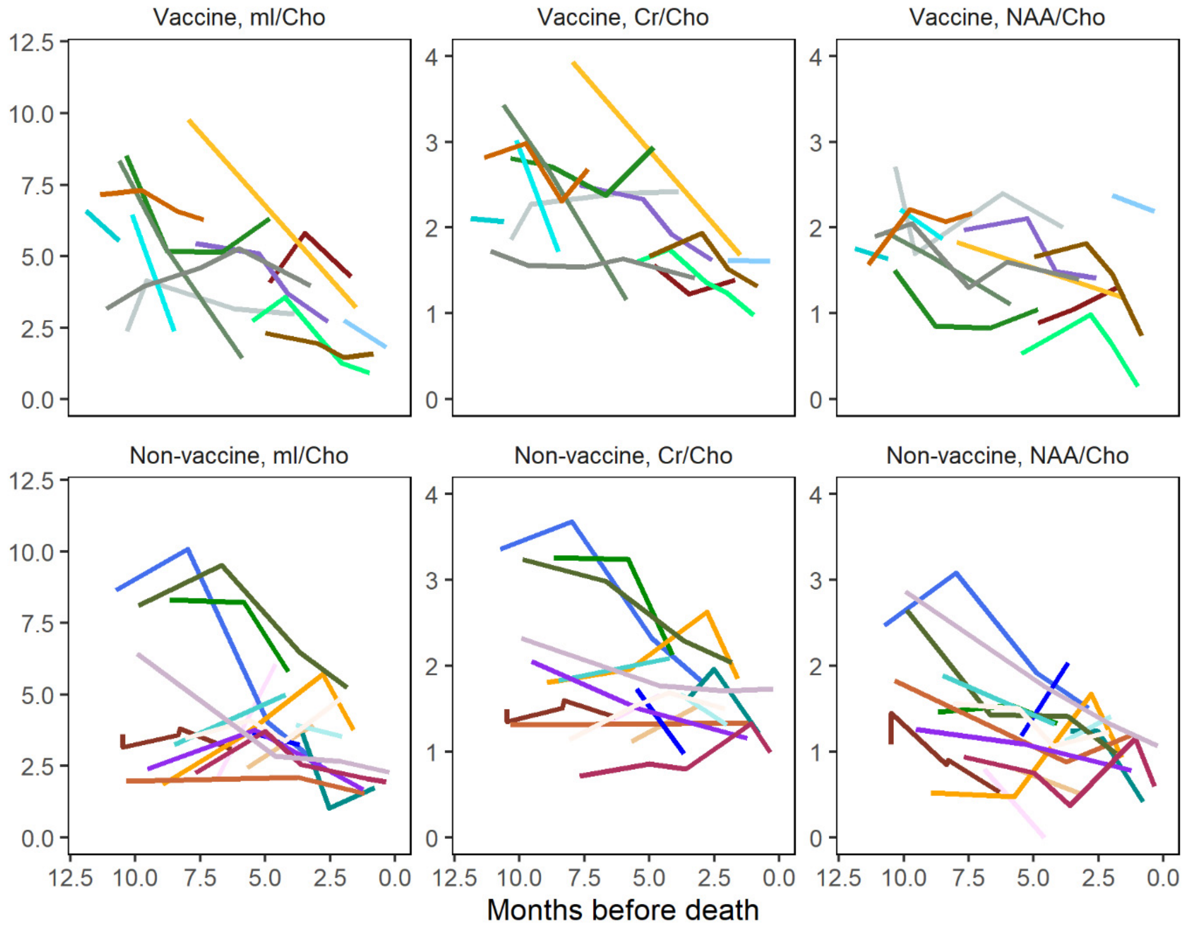

Figure S5. Terminal decline (relative to date of death, X=0) for metabolite ratios of interest measured within 12 months of death, excluding scans at diagnosis: myo-Inositol/choline (left column), creatine/choline (middle), NAA/choline (right column). .

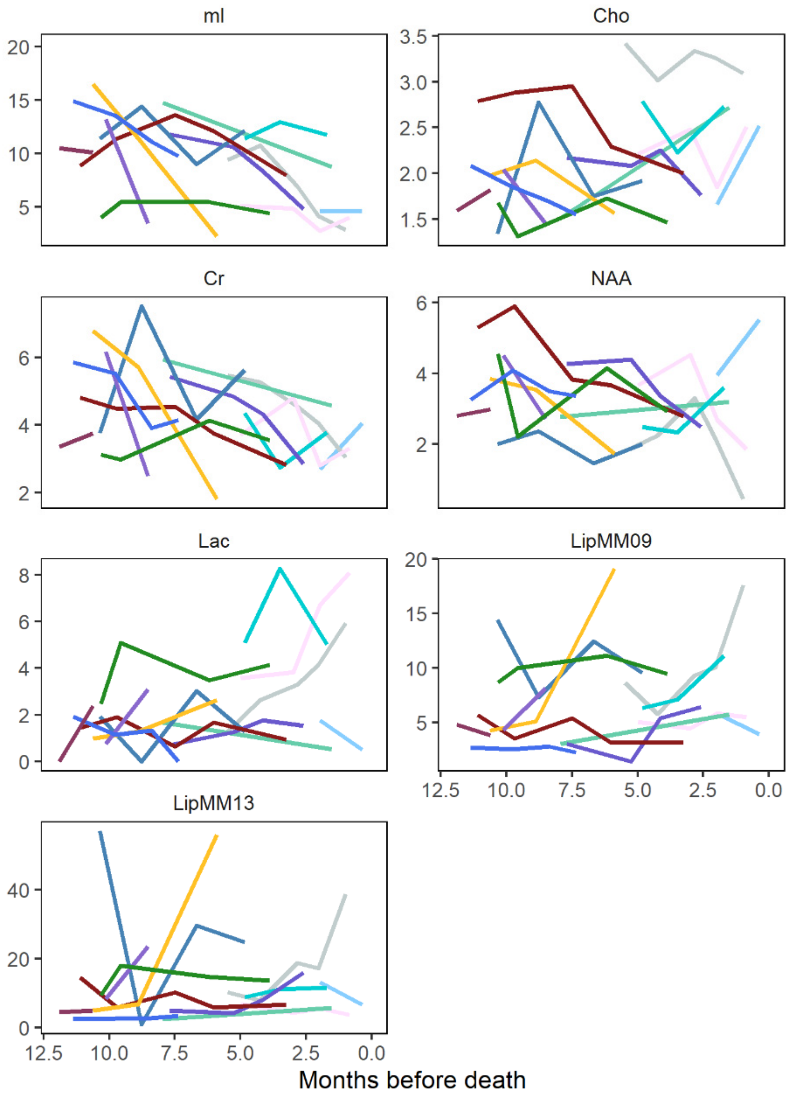

Figure S6. Terminal change (relative to date of death) for individual metabolites (mmol/kg), **Vaccine Cohort** (58 scans in 14 patients, limited to within 12 months of death). mI = myo-inositol; Cho = choline; Cr = creatine; NAA = N-acetyl-aspartate; Lac = lactate; LipMM09 = lipid intensity at 0.9 ppm, terminal -CH3; LipMM13 = lipid intensity at 1.3 ppm, terminal –CH2;.

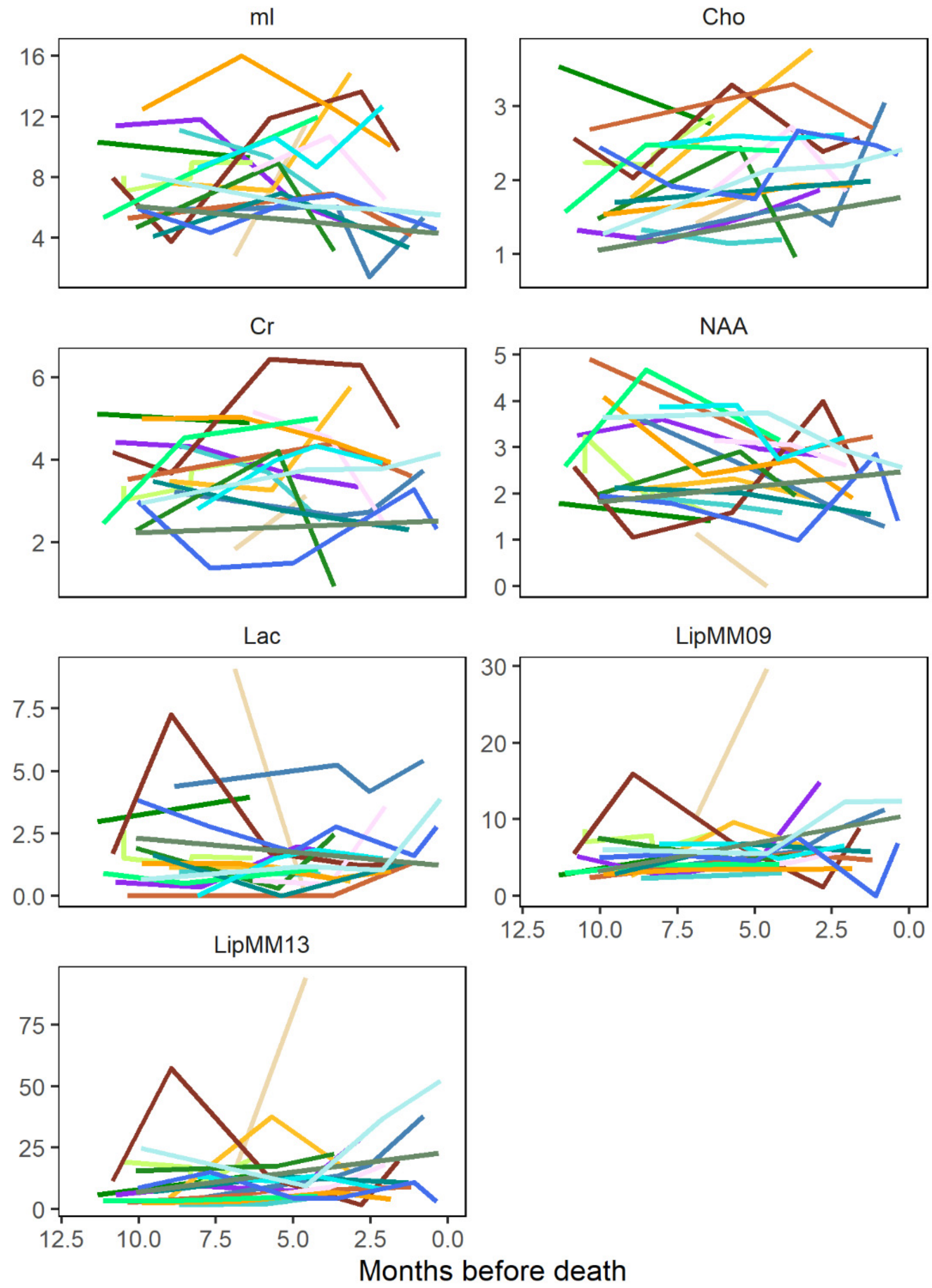

Figure S7. Terminal change (relative to date of death) for individual metabolites (mmol/kg), **Non-vaccine Cohort** (87 scans in 32 patients, limited to within 12 months of death).
